# Optimization of Ionic Matrix Deposition to Increase the Performance of MALDI‐Based Spatial N‐Glycomics

**DOI:** 10.1002/jms.70053

**Published:** 2026-03-22

**Authors:** Heidi Vandyk, Christopher Anderton, Hak Joo Lee, Kumar Sharma, Dušan Veličković

**Affiliations:** ^1^ Environmental Molecular Sciences Division Pacific Northwest National Laboratory Richland Washington USA; ^2^ Center for Renal Precision Medicine, Division of Nephrology, Department of Medicine The University of Texas Health San Antonio Texas USA

**Keywords:** dimethylaniline, imaging, ionic matrix, N‐glycan, sialic acid

## Abstract

Ionic matrices for matrix‐assisted laser desorption/ionization (MALDI) have demonstrated superior performance compared to traditional matrices for profiling and screening of carbohydrate composition. However, their application in MALDI‐MS imaging of N‐glycans in tissues remains elusive, in part due to suboptimal matrix application conditions. In this study, we optimized 2,5‐dihydroxybenzoic acid/N, N‐dimethylaniline (DHB/DMA) ionic matrix spray conditions to enhance the sensitivity of spotted N‐glycan standards; however, these conditions were not suitable for analyzing endogenous N‐glycans in tissue. To address this, we refined the composition of the ionic matrix by replacing DHB with α‐cyano‐4‐hydroxycinnamic acid (CHCA) and DMA with its hydrochloride salt (DMA·HCl), resulting in superior performance compared to the CHCA matrix, which is the most widely used for spatial N‐glycomics. This method enhanced the sensitivity for detecting diverse N‐glycan types, including low‐abundance sialic acid glycans, while maintaining their endogenous location—a key limitation of liquid‐state DMA.

## 1 Introduction

Ionic liquid matrices (ILMs), particularly those composed of N, N‐dimethylaniline (DMA) and 2,5‐dihydroxybenzoic acid (DHB) or α‐cyano‐4‐hydroxycinnamic acid (CHCA), are recognized for their remarkable efficiency in matrix‐assisted laser desorption/ionization (MALDI) analyses of various carbohydrates, including small sugars, glycans, as well as neutral, amino, and acidic oligosaccharides [1, 2, 3, 4, 5, 6, 7]. These ILMs are hypothesized to outperform conventional crystalline solid matrices due to the unique properties of the aromatic acid/aniline salt, which exhibits superior absorption and redistribution of laser energy [7, 8]. Notably, carbohydrate analyses using ionic matrices containing DMA demonstrate substantial improvements in sensitivity, which often surpass those achieved with traditional matrices such as CHCA or DHB in dry‐droplet methods [7]. Additionally, ILMs generate highly homogeneous matrix depositions with a solid‐solution‐like appearance, ensuring uniform MALDI signal distribution across the sample and enhancing reproducibility. Conversely, crystalline matrices can form localized “hot spots” of analyte‐ion production, negatively affecting reproducibility and complicating sample analysis [3].

One significant advantage of using ionic matrices is their ability to preserve labile acidic groups on glycans, such as sulfates and sialic acids [9]. This feature is particularly critical for imaging N‐glycans in biological tissues, where sialic acids—key modulators of numerous physiological and pathological processes [10]—are often lost during conventional MALDI workflows [11, 12]. In an effort to overcome this challenge, additional derivatization or protective strategies have been reported to preserve these labile groups—steps that are unnecessary with the use of ionic matrices [13, 14].

Despite their distinct advantages, the application of ILMs in MALDI‐MS imaging for carbohydrate analysis remains extremely limited. To date, only three studies have employed ILMs for glycan imaging. The Rogniaux group pioneered the use of ionic matrices for hemicellulose analysis in cereals [15, 16]. Additional reports include their application for N‐glycan detection directly from thin‐layer chromatography (TLC) plates [17] and for imaging N‐glycans from brain tissue [18]. However, these studies used different matrix deposition techniques, including vibrational vaporization, automated spraying, and airbrushing. This inconsistency highlights the need for developing standardized workflows to advance the use of ILMs in MALDI‐MS imaging.

The limited adoption of ILMs in imaging applications is likely due to the noncomplementary physicochemical behavior of the two MALDI matrix components during deposition. This results in the formation of moisturized solid solution deposits, which can compromise glycan localization and spatial accuracy—key factors for generating highly precise and detailed molecular images. In fact, the spatial resolutions reported in the three studies utilizing ionic matrices were all ≥ 100 μm [16, 17, 18]. These resolutions are insufficient to capture single‐cell processes, and they fall short of the capabilities of modern glycan MS imaging instrumentation, which regularly achieve resolutions of 10 μm to produce sharp ion images that reliably reveal the cellular origin of glycans [19]. Here, we tackle the challenge of optimizing ionic matrix application for MALDI‐MS imaging to fully leverage its ability to enhance glycan sensitivity and enable its use in high‐resolution imaging modalities.

## 2 Materials and Methods

### 2.1 N‐Glycan Standard Preparation

Oligomanose N‐linked oligosaccharide with five mannosyl residues (Man‐5 glycan) and monosialyated biantennary oligosaccharide (A1 glycan) purchased from QA‐Bio were dissolved in 50% acetonitrile to a final concentration of 500 fmol/μL and 5 pmol/μL, respectively. One microliter of each standard was then spotted onto indium tin oxide (ITO)‐coated slides at two places and allowed to dry entirely before MALDI matrix application.

### 2.2 Tissue Preparation Before Matrix Application

The IACUC of UT Health San Antonio approved the animal experiment (Protocol #: 20170168AR). 8–10 weeks db/db mice with type 2 diabetes (Strain #: 000664, Jackson Laboratory) were randomized, then the mice received vehicle or 10 mg/kg of MTAP inhibitor by drinking water for 8 weeks. The kidney was harvested, fixed with 10% neutral buffered formalin, and then embedded into paraffin blocks. FFPE‐preserved human kidney nephrectomy tissue was acquired at the University of Michigan and sent to Pacific Northwest National Laboratory. Tissues were sectioned at 2 μm and serial sections were mounted on indium tin oxide (ITO)‐coated slides [20]. Slides were then heated at 60°C, dewaxed, and rehydrated before antigen retrieval in boiling citraconic buffer for 30 min, as described previously [19]. PNGase‐F (N‐Zyme Scientifics, 100 μg/mL) was sprayed onto the slides using an automated sprayer (M5, HTX Technologies) at a 25 μL/min flow rate, with 15 passes, in a crisscross pattern, a 1200 mm/min spray head velocity, and a 3.0‐mm track spacing. Slides were then placed in preheated humidity incubation chambers filled with a saturated water solution of KNO_3_ to maintain 89% relative humidity [19] and incubated for 2 h at 37°C before MALDI matrix application.

### 2.3 MALDI Matrix Preparation and Application

MALDI matrices α‐cyano‐4‐hydroxycinnamic acid (CHCA, Catalog Number 70990, Sigma), 2,5‐dihydroxybenzoic acid (2,5‐DHB, Catalog Number 85707, Sigma), together with boosting agents N, N‐dimethylaniline (DMA, Catalog Number N515124, Sigma) and N, N‐dimethylaniline hydrochloride (DMA·HCl, Batch No. B25BS06272, BOC Sciences), were used in MALDI matrix preparation.

For all experiments detailed in the manuscript, the MALDI matrix was sprayed using an automated sprayer (M5, HTX Technologies). For the CHCA (7 mg/mL in 50% acetonitrile (AN) and 0.1% trifluoroacetic acid in water [v/v]) application, previously established settings were used: 100 μL/min, 10 passes, a crisscross pattern, a velocity of 1300 mm/min, at 80°C, and 3.0‐mm track spacing [19]. For the DHB application, the protocol reported by Everest‐Dass was used, where 20 mg/mL of DHB (0.1% TFA, 1 mM NaCl in water) was sprayed at 50 μL/min, 16 passes, a crisscross pattern, a velocity of 800 mm/min, at 65°C, and 2‐mm track spacing [21]. The DHB/DMA ionic matrix was prepared by making the final concentration of 100 mg/mL of 2,5‐dihydroxybenzoic acid (DHB) and 20 μL/mL of DMA in 50% AN according to established protocol [1]. Different HTX settings were used for spraying DHB/DMA, as listed in Table 1. CHCA/DMA ionic matrix was prepared by making the final concentration of 7 mg/mL of CHCA and 1.4 mg/mL of DMA in 50% AN and sprayed using the same parameters used for CHCA spraying: 100 μL/min, 10 passes, a crisscross pattern, a velocity of 1300 mm/min, at 70°C, and 3.0‐mm track spacing. Alternatively, CHCA was sprayed before DMA, or DMA was sprayed before CHCA, using the same spraying parameters as used for CHCA application. CHCA/DMA·HCl binary matrix was prepared by making the final concentration of 7 mg/mL of CHCA and 1.4 mg/mL of DMA·HCl in 50% AN and sprayed using the same parameters used for CHCA spraying: 100 μL/min, 10 passes, a crisscross pattern, a velocity of 1300 mm/min, at 70°C, and 3.0‐mm track spacing.

**TABLE 1:** Signal intensity of Man5 standard and appearance of the ionic MALDI matrix in different spraying conditions. Each horizontal line represents 2 × 10^7^ increment in signal intensity.

Man5 signal [6] intensity [4] (10 ^7^ au) [2]	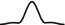	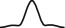	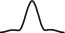	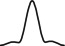	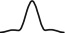	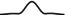	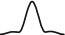	
Optical images	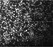	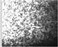	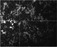	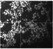	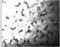	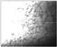	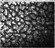	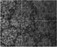
Description	Ultraslow moderate T		Ultraslow low T	Ultraslow high T	Slow moderate T		Fast low T	Ultrafast high T
FR (mL/min)	0.01	0.01	0.01	0.01	0.05	0.05	0.1	0.25
V (mm/min)	600	600	600	600	800	800	1200	1000
LS (mm)	1	1	1	1	1	1	2.5	3
NP	3	6	6	6	8	16	3	2
T (°C)	65	65	30	80	65	65	30	85
D (μg/mm^2^)	5	10	10	10	25	50	10	17

Abbreviations: D, density of matrix; FR, flow rate; LS, line spacing; NP, number of passes; T, temperature; V, velocity.

### 2.4 MALDI MSI Analysis

All analyses of N‐glycan standards were performed using a 15 Telsa SolariX Fourier‐transform ion cyclotron resonance (FTICR) mass spectrometer (Bruker Daltonics, Bremen, Germany) equipped with a dual ESI/MALDI ion source and a Smartbeam II Nd:YAG (355 nm) laser. The instrument was operated in positive‐ion mode over an *m/z* range of 600–2000, with an estimated resolving power of 320 000 at *m/z* 400. All tissue analyses were performed using timsTOF Flex (Bruker Daltonics) operated in positive ion mode over the *m/z* range 900–4000, using a 20‐μm step size, and the TIMSin pressure was adjusted to ∼1.8 mbar.

## 3 Results and Discussion

### 3.1 DHB/DMA as an Ionic Matrix for Analyzing N‐Glycan Standards

**FIGURE 1:**
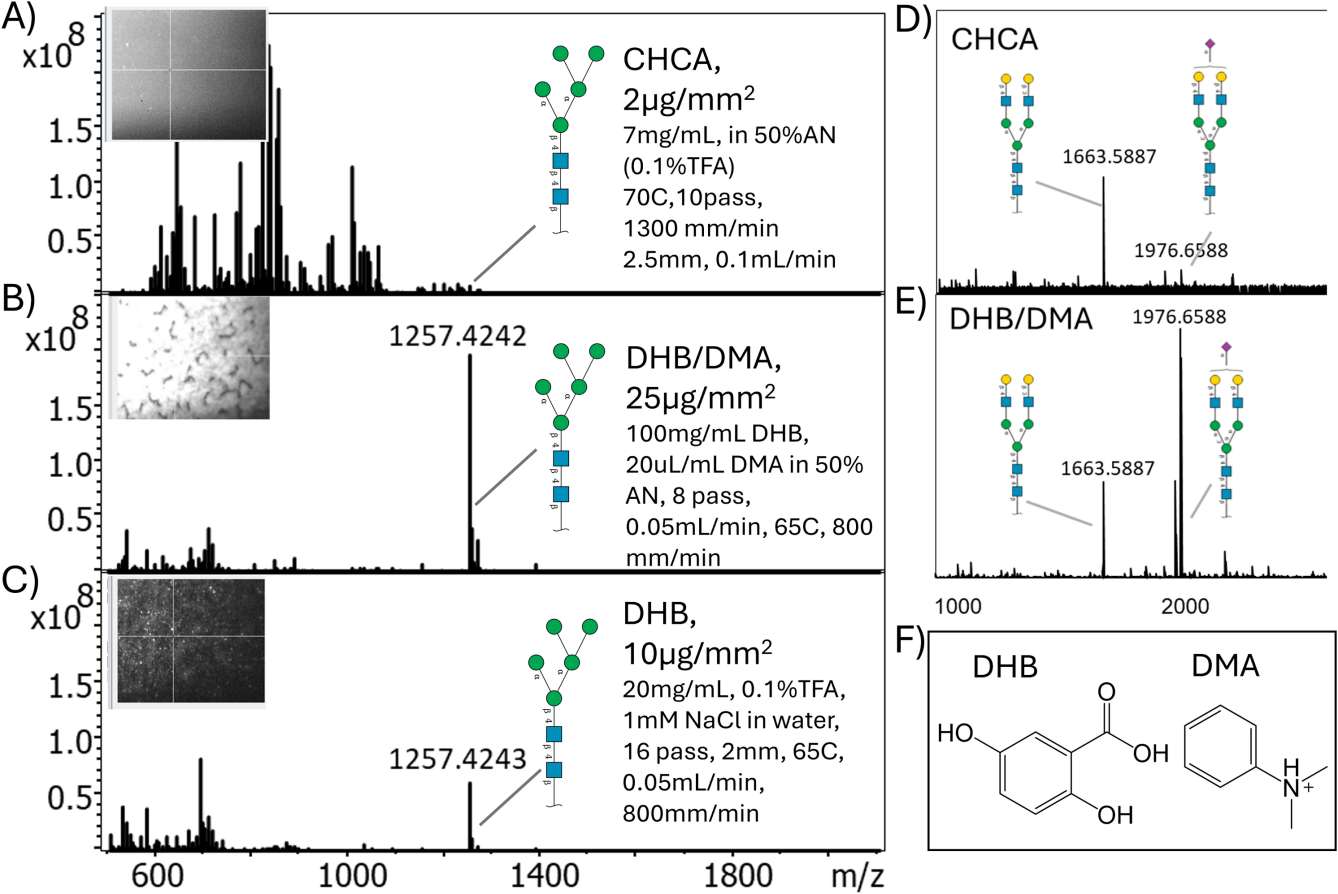
Boosting of N‐glycan signal intensity and preserving sialic acid glycan integrity using ILM in MALDI analysis. 500 fmol of Man‐5 N‐glycan MALDI mass spectrum after (A) application of CHCA according to the protocol described in our most recent publication [22], (B) DHB/DMA, and (C) DHB according to the protocol described by Everest‐Dass et al. [22] Inserts show the appearance of MALDI matrix crystals viewed under the MALDI camera. 5 pmol of A1 N‐glycan MALDI mass spectrum in (D) CHCA and (E) DHB/DMA MALDI matrix. (F) Chemical structure of ILM (DHB/DMA).

Figure 1 highlights the two primary advantages of integrating ILM into N‐glycan imaging analysis. Firstly, ILM significantly enhances signal intensity, thereby improving the limit of detection (LOD) for N‐glycans (Figure 1A–C). Secondly, it effectively preserves sialic acid decorations, ensuring greater structural integrity of these labile molecules during analysis (Figure 1D– E).

When compared to the conventional CHCA MALDI matrix method—where 500 fmol of the Man 5 standard remains undetectable—the ILM matrix demonstrates a remarkable fivefold signal increase relative to the traditional DHB MALDI matrix application for N‐glycan analysis, with the N‐glycan signal being the most abundant peak. Furthermore, the analysis of sialic acid glycans using the conventional CHCA matrix results in significant in‐source fragmentation (~85%), with the base peak corresponding to the glycan without its sialic acid (*m/z* 1663.588). In contrast, the DHB/DMA matrix preserves the glycan's sialic acid, making it the most dominant peak (*m/z* 1976.658), with only a minor fraction (~25%) experiencing in‐source fragmentation. This preservation phenomenon is likely attributed to the efficient absorption of laser energy by DMA, which exhibits a pronounced absorption peak near 298 nm (Supporting Figure S1). This strong absorption arises from the conjugated system of the benzene ring and the lone pair of electrons on the nitrogen atom, enhancing its interaction with the laser energy.

In the initial experiments, DHB and CHCA were applied according to established, widely used protocols to produce fine crystal deposits suitable for MALDI MSI of N‐glycans (Figure 1A,C, respectively). Conversely, the initial application of the DHB/DMA matrix was performed under sprayer settings delivering 25 μg/mm^2^ across eight passes. This density, while on the higher end of traditional MALDI MSI matrix applications, aligns with ionic matrix densities utilized by the Rogniaux group for oligosaccharide imaging [16]. Results show that DHB/DMA significantly boosted analytical sensitivity compared to DHB and CHCA. However, the MALDI deposits formed under these settings were nonhomogeneous, exhibiting large chunks and aggregates (Figure 1B), which are unsuitable for MALDI MSI applications.

To overcome this challenge, sprayer parameters were varied to assess the effects of application speed, flow rate, and temperature on MALDI deposit morphology (Table 1). The resulting applications spanned matrix densities ranging from 5 to 50 μg/mm^2^, while the matrix concentration was consistently maintained at 100 mg/mL DHB and 20 μL/mL DMA, in line with standard concentrations used for all DHB/DMA protocols. Across all settings, a prominent signal was detected for the 500 fmol Man5 standard, except in the ultrafast application (FR 0.25 mL/min, V 1000 mm/min). Notably, this ultrafast application produced intense signal suppression, eliminating glycan signal detection despite being previously reported as effective [17].

Regardless of the tested settings, MALDI deposits remained nonhomogeneous, making them unsuitable for MALDI‐MSI. This was further confirmed by tissue analysis, in which DHB/DMA application settings associated with high sensitivity (ultraslow application at low temperature) from the standard led to massive signal suppression and proved far less effective than traditional CHCA matrix applications (Supporting Figure S2). These findings demonstrate that simply translating MALDI matrix composition and application spray settings from dry‐droplet standards to tissue analysis is neither straightforward nor directly applicable.

### 3.2 CHCA/DMA as an Ionic Matrix for Tissue Analysis

To address the limitations observed with DHB/DMA matrices, we explored an alternative strategy by replacing DHB with CHCA in the ionic matrices. CHCA, when combined with DMA, has been demonstrated to produce solid ionic matrices, unlike the amorphous appearance seen with DHB/DMA [23]. Additionally, CHCA is known for forming extremely fine, homogeneous MALDI crystals, making it the preferred matrix for most laboratories performing spatial N‐glycomics.

**FIGURE 2:**
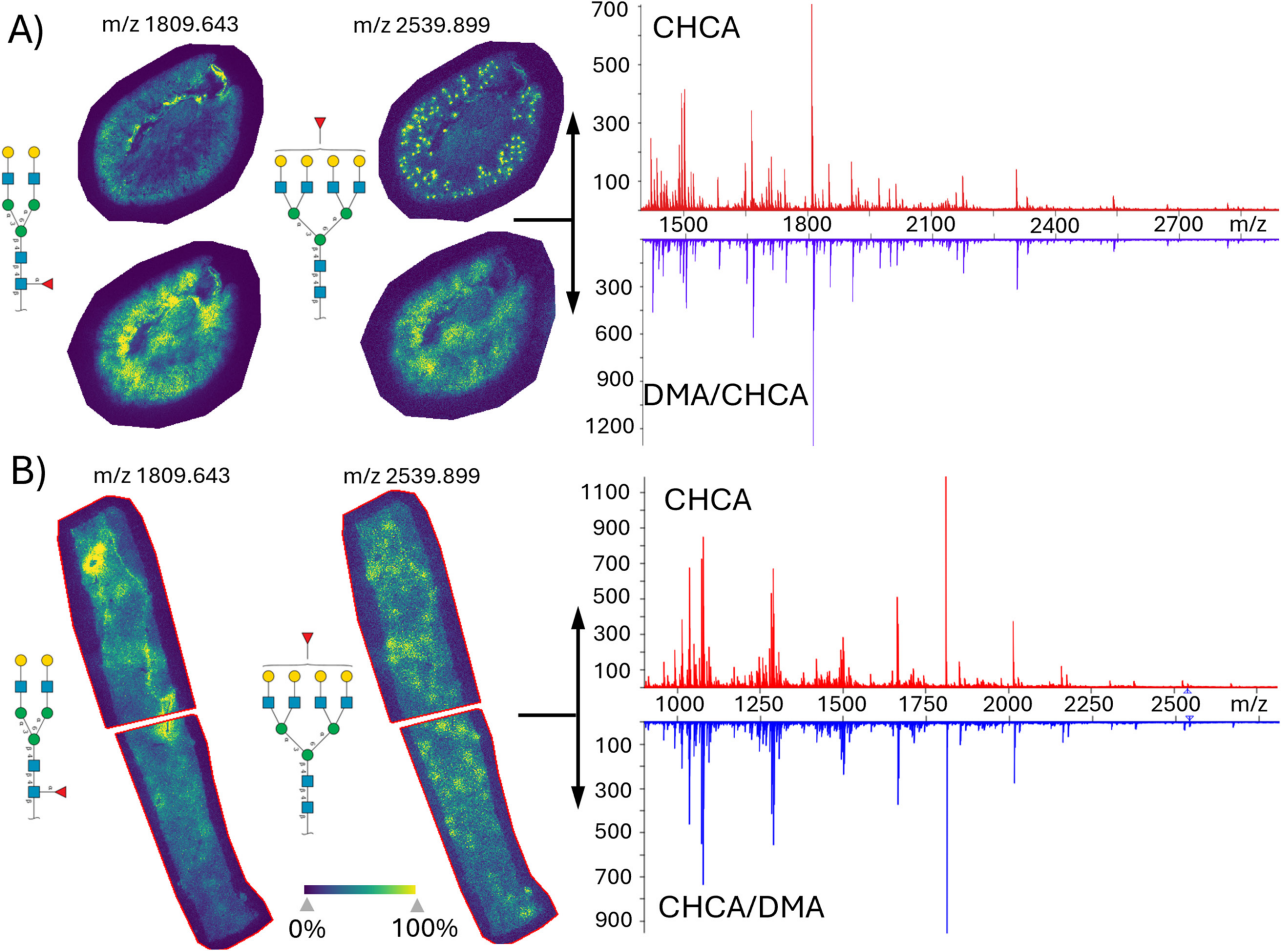
MALDI mass spectra and N‐glycan ion images obtained in conventional CHCA and ionic DMA/CHCA MALDI matrix. (A) In the DMA/CHCA MALDI matrix, DMA was first sprayed, followed by CHCA. (B) In the CHCA/DMA MALDI matrix application, CHCA was sprayed first, and the upper half of the tissue was analyzed. Subsequently, DMA was sprayed, and the lower half of the tissue was analyzed. Mouse kidney section (A) and human nephrectomy section (B) were analyzed at a 20‐μm step size.

To evaluate the efficacy of CHCA/DMA matrices, we tested three distinct methods of application using an HTX sprayer: (i) spraying DMA and CHCA as a premixed solution, (ii) sequential application of DMA followed by CHCA spraying, and (iii) sequential application of CHCA followed by DMA spraying. The performance of these methods was compared to the standardized CHCA application protocol (Figure 2).

Initial testing with premixed DMA and CHCA showed significant signal suppression, similar to observations with the DMA/DHB matrix. However, distinct findings emerged in the sequential application. Notably, the signal intensity of all N‐glycans increased by approximately 200% when DMA was applied before CHCA. On the downside, delocalization effects were observed, exemplified by N‐glycans characteristic of the kidney glomerulus, which displayed a relatively diffuse distribution in the DMA/CHCA application (Figure 2A). This delocalization could be attributed to the application of more passes (e.g., solvent volume) in the sequential settings, or to DMA being in a liquid‐oily form, or to both. In contrast, the sequential application of CHCA followed by DMA did not cause signal delocalization. We speculate that CHCA had already formed co‐crystals with the glycans at the tissue surface, making subsequent solvent layers irrelevant for crystal size modification. However, in this configuration, DMA did not enhance the sensitivity of detected N‐glycans (Figure 2B). These findings suggest that DMA must interact with the glycan before its incorporation into CHCA crystals to leverage the signal boosting effect of the ionic matrix.

### 3.3 Evaluation of CHCA and DMA·HCl as a Binary Matrix for Tissue Imaging

Because none of the CHCA/DMA combinations produced the desired results, and given that DMA is a liquid, we chose to utilize the hydrochloride (HCl) salt of DMA—a solid—to avoid liquid spreading across the tissue surface following matrix solvent evaporation. In this approach, we intentionally aimed to leverage the benefits of the solid form of DMA rather than the ionic matrix properties of its mixture with CHCA. Notably, DMA·HCl, being the conjugate acid of DMA, means its mixture with CHCA cannot strictly be classified as an ionic matrix.

**FIGURE 3:**
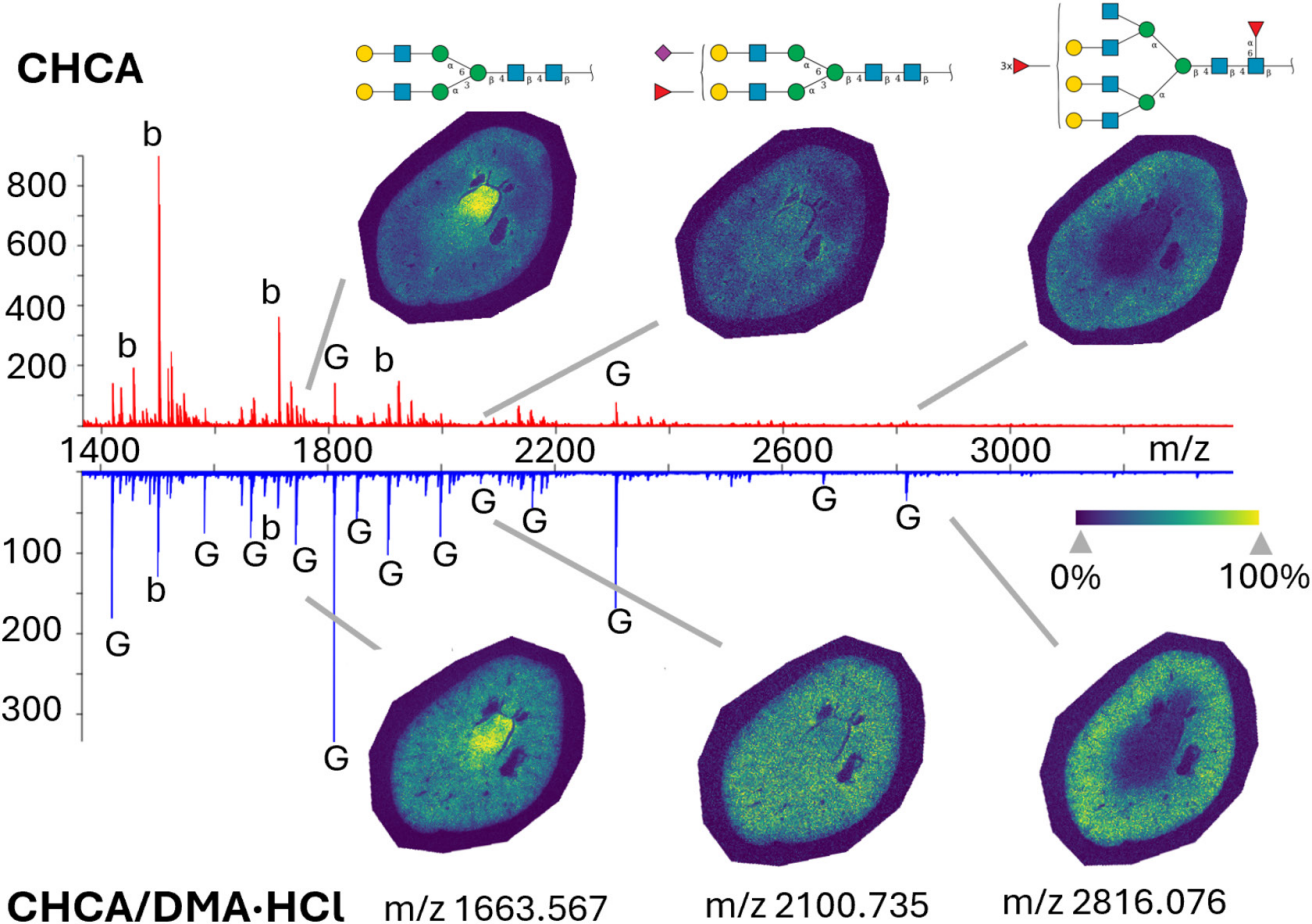
MALDI mass spectra and N‐glycan ion images from mouse kidney obtained using conventional CHCA and CHCA/DMA·HCl mixture as MALDI matrix. b, background signal; G, glycan signal.

When sprayed as a mixture, CHCA/DMA·HCl formed a homogeneous, dry crystalline layer over both the ITO slide and tissue, closely resembling the appearance of CHCA alone (Supporting Figure S3). However, the MALDI mass spectra and results obtained with the two matrices are significantly different. While the conventional CHCA application resulted in intense background peaks (labeled with “b” in Figure 3), the dominant signals in the CHCA/DMA·HCl applications were N‐glycan peaks (labeled with “G” in Figure 3). This contrast was particularly pronounced in tissues with decreased N‐glycan abundance, as demonstrated in Figure 3, where several low‐abundant sialic acid N‐glycans were newly annotated (list of all detected N‐glycans is provided in Supporting Table S1 and ion images can be viewed in the associated METASPACE link). Notably, although the total ion counts in this binary matrix decreased, N‐glycan signals increased overall by up to 200%. This enhancement was observed across various glycan types, including complex, fucosylated, and, most importantly, low abundance sialylated glycans (Figure 3). Although two peaks were annotated as sulfated N‐glycans (Supporting Table S1), isobaric nonsulfated species were assigned with higher confidence, leaving it unclear whether this binary matrix preserves sulfated glycans, as we were unable to detect them with confidence in our datasets. Crucially, signal localization remained consistent with the controlled CHCA application, indicating no delocalization when CHCA/DMA·HCl is sprayed together, suggesting the potential of CHCA/DMA·HCl to enhance signal in tissues with low glycan abundance while still providing reliable, high‐resolution images.

## 4 Conclusions

We demonstrated the benefits of boosting sensitivity and preserving sialic acid in N‐glycan standards by using the ionic DHB/DMA MALDI matrix. However, our results showed that even under optimal standard‐analysis conditions, a pronounced suppression effect was observed using this ionic matrix when endogenous N‐glycans were analyzed from tissue. Through optimization of ionic matrix compositions and application conditions, we eventually identified a novel combination of CHCA and DMA·HCl that significantly boosts N‐glycan signal intensities by up to 200% in imaging modality, without compromising N‐glycans' native molecular localization. Although more extensive physicochemical characterization of the CHCA/DMA·HCl binary matrix is needed to fully elucidate the basis of its N‐glycan–enhancing effects in MALDI imaging, it is likely that the DMA·HCl/CHCA combination outperforms DMA/CHCA because the more polar, protonated DMA·HCl integrates more effectively into the acidic CHCA matrix during spraying, promoting more homogeneous co‐crystallization and consequently more efficient energy transfer. Differences in their UV absorption profiles (Supporting Figure S1) may further contribute to the distinct behavior of these matrices within the complex MALDI desorption/ionization process. The key advantage of using DMA·HCl is its solid form, which bypasses the delocalization limitations observed with liquid DMA as a counterion. This finding provides a promising avenue for applying ionic matrices in N‐glycan research, particularly for the detection and analysis of low‐abundance and labile glycans at the cellular resolution, as we observed for many fucosylated and sialylated glycans. While analyses of additional sample types and glycan classes may clarify whether this approach also protects sulfated glycans, which we were unable to detect in the kidney samples analyzed.

It is important to acknowledge that we also explored alternative matrix deposition techniques of this binary matrix, such as nebulization and sublimation, but these approaches proved ineffective. Nebulization resulted in heterogeneous deposition with large amorphous crystals (Supporting Figure S4), while DMA decomposed under the high temperature required for sublimation and was subsequently undetected in the MALDI mass spectrum (Supporting Figure S5). Future investigations could consider alternative methods, such as low‐temperature, high‐pressure evaporation systems [23], to further optimize ionic matrix application for MALDI imaging of N‐glycans.

## Author Contributions

**Dušan Veličković:** conceptualization, resources, funding, data curation, writing original draft, project management, and formal analysis. **Heidi Vandyk:** MALDI MSI sample preparation, running the instrument, investigation, and formal analysis. **Christopher Anderton:** resources, reviewing, and editing the manuscript. **Hak Joo Lee:** FFPE sample preparation. **Kumar Sharma:** resources.

## Funding

This work was supported by the National Institute of General Medical Sciences (R35GM156251).

## Supporting information


**Figure S1:** UV absorption spectra (200–400 nm) of DMA and DMA·HCl in 50% acetonitrile as solvent.
**Figure S2:** Comparison of MALDI MS spectra from kidney nephrectomy after spraying released N‐glycans with DHB/DMA ionic matrix and CHCA MALDI matrix. A huge suppression of N‐glycans in DHB/DMA conditions is observed.
**Figure S3:** Appearance of deposited CHCA and CHCA/DMA·HCl matrices under the 20× objective on the light microscope
**Figure S4:** Comparison of the appearance of nebulized DMA/DHB matrix and sprayed CHCA matrix applied over N‑glycan standards on an ITO glass slide. (A) DMA/DHB applied with 52 cycles, 25% spray power, 30% modulation, 2‐s spray time, 30‐s incubation, and 60‐s dry time. (B) DMA/DHB applied with 6 cycles, 20% spray power, 25% modulation, 2‐s spray time, 30‐s incubation, and 60‐s dry time. Large, amorphous DMA/DHB crystals are observed under both application conditions.
**Figure S5:** Effects of CHCA and CHCA/DMA sublimation on MALDI matrix signals, N‑glycan signals, and N‑glycan imaging. (A) MALDI spectra in the low m/z range for CHCA and CHCA/DMA after spraying and after sublimation. Sublimation of CHCA produces a strong unknown peak at m/z 195.1, whereas CHCA/DMA sublimation does not show a detectable DMA‐related signal. (B) The A1 sialylated N‑glycan is not preserved following CHCA/DMA sublimation. (C) CHCA/DMA sublimation yields images with similar quality and intensity to those obtained when only CHCA is sublimed.

**Table S1:** Supporting information.

## Data Availability

MALDI‐MSI data are available on METASPACE. Project: https://metaspace2020.org/project/velickovic‐2026_Ionic.
