# Supplementary material for: Optimization of Ionic Matrix Deposition to Increase the Performance of MALDI‐Based Spatial N‐Glycomics
Source: J Mass Spectrom. 2026 Mar 22;61(4):e70053. doi: 10.1002/jms.70053 (PMC13006149; doi:10.1002/jms.70053)
Supplement: Supplementary file 1 — Figure S1: UV absorption spectra (200–400 nm) of DMA and DMA·HCl in 50% acetonitrile as solvent. Figure S2: Comparison of MALDI MS spectra from kidney nephrectomy after spraying released N‐glycans with DHB/DMA ionic matrix and CHCA MALDI matrix. A huge suppression of N‐glycans in DHB/DMA conditions is observed. Figure S3: Appearance of deposited CHCA and CHCA/DMA·HCl matrices under the 20× objective on the light microscope Figure S4: Comparison of the appearance of nebulized DMA/DHB matrix and sprayed CHCA matrix applied over N‑glycan standards on an ITO glass slide. (A) DMA/DHB applied with 52 cycles, 25% spray power, 30% modulation, 2‐s spray time, 30‐s incubation, and 60‐s dry time. (B) DMA/DHB applied with 6 cycles, 20% spray power, 25% modulation, 2‐s spray time, 30‐s incubation, and 60‐s dry time. Large, amorphous DMA/DHB crystals are observed under both application conditions. Figure S5: Effects of CHCA and CHCA/DMA sublimation on MALDI matrix signals, N‑glycan signals, and N‑glycan imaging. (A) MALDI spectra in the low m/z range for CHCA and CHCA/DMA after spraying and after sublimation. Sublimation of CHCA produces a strong unknown peak at m/z 195.1, whereas CHCA/DMA sublimation does not show a detectable DMA‐related signal. (B) The A1 sialylated N‑glycan is not preserved following CHCA/DMA sublimation. (C) CHCA/DMA sublimation yields images with similar quality and intensity to those obtained when only CHCA is sublimed. [file JMS-61-e70053-s001.docx]

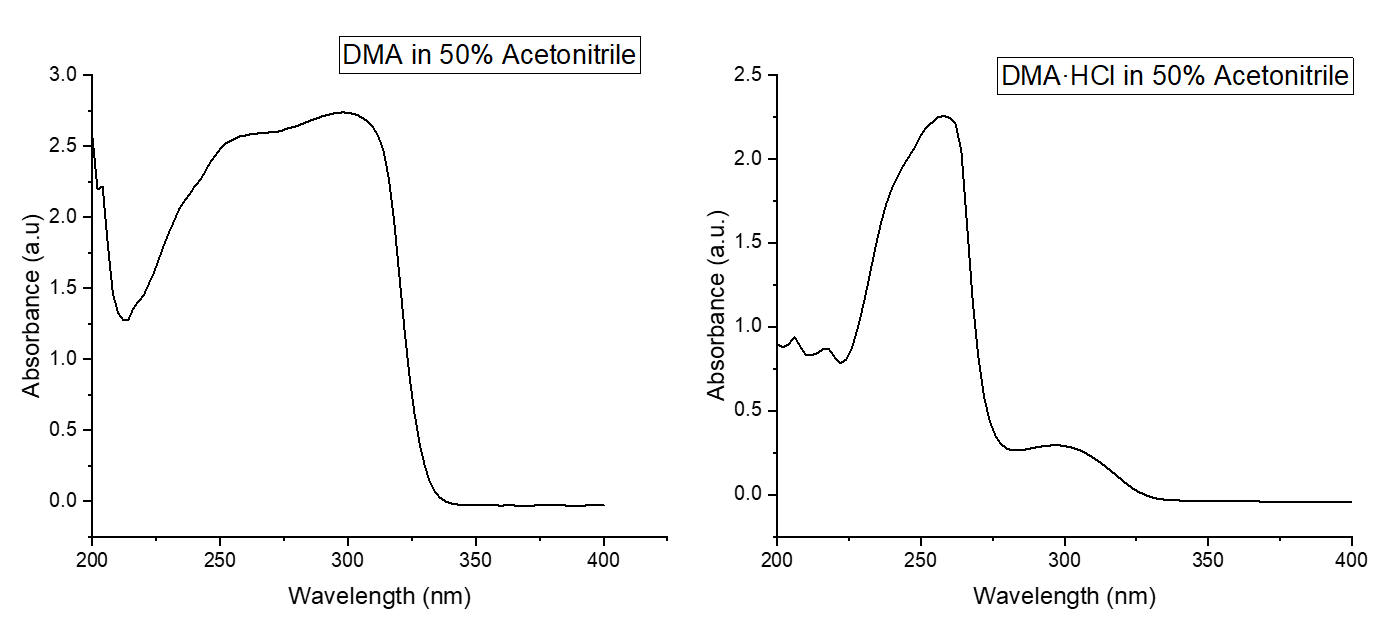


**Supporting Figure S1.** UV absorption spectra (200-400 nm) of DMA and DMA·HCl in 50% acetonitrile as solvent.


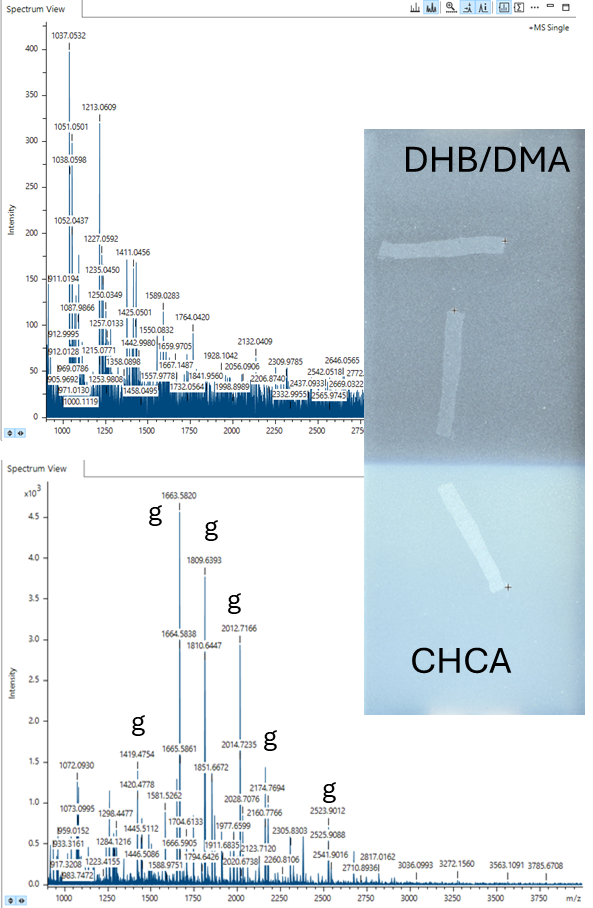


**Supporting Figure S2.** Comparison of MALDI MS spectra from kidney nephrectomy after spraying released N-glycans with DHB/DMA ionic matrix and CHCA MALDI matrix. A huge suppression of N-glycans in DHB/DMA conditions is observed.


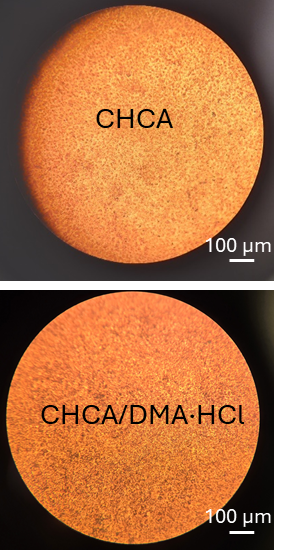


**Supporting Figure S3**. Appearance of deposited CHCA and CHCA/DMA·HCl matrices under the 20X objective on the light microscope.


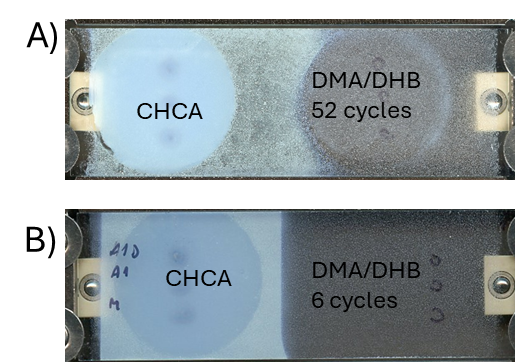


**Supporting Figure S4.** Comparison of the appearance of nebulized DMA/DHB matrix and sprayed CHCA matrix applied over N‑glycan standards on an ITO glass slide. A) DMA/DHB applied with 52 cycles, 25% spray power, 30% modulation, 2 s spray time, 30 s incubation, and 60 s dry time. B) DMA/DHB applied with 6 cycles, 20% spray power, 25% modulation, 2 s spray time, 30 s incubation, and 60 s dry time. Large, amorphous DMA/DHB crystals are observed under both application conditions.


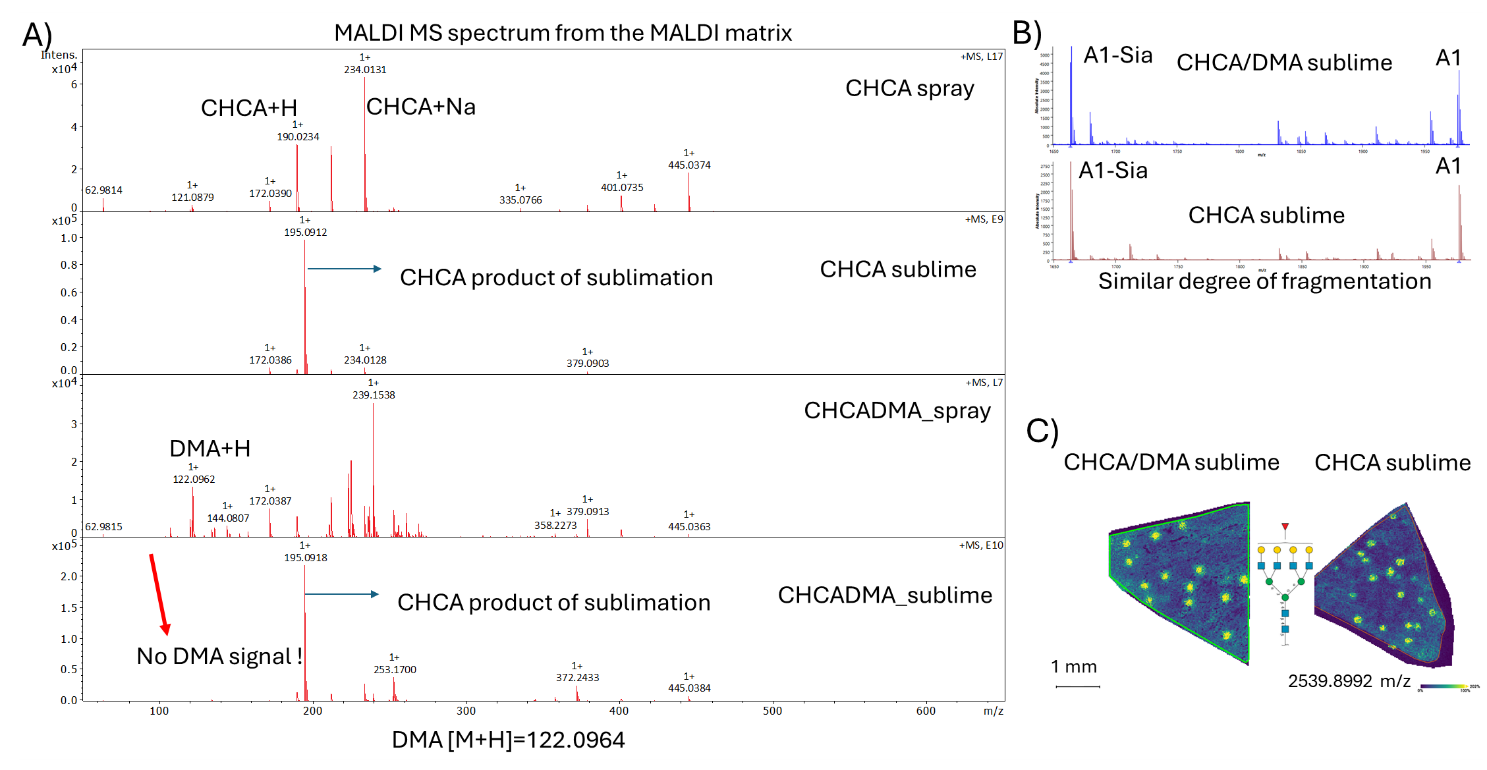


**Supporting Figure S5.** Effects of CHCA and CHCA/DMA sublimation on MALDI matrix signals, N‑glycan signals, and N‑glycan imaging. A) MALDI spectra in the low m/z range for CHCA and CHCA/DMA after spraying and after sublimation. Sublimation of CHCA produces a strong unknown peak at m/z 195.1, whereas CHCA/DMA sublimation does not show a detectable DMA-related signal. B) The A1 sialylated N‑glycan is not preserved following CHCA/DMA sublimation. C) CHCA/DMA sublimation yields images with similar quality and intensity to those obtained when only CHCA is sublimed.
